# Older Adults’ Adaptation to Smart Mobility Technology: Bridging the Digital Divide for Independent Mobility

**DOI:** 10.1093/geroni/igaf122.1995

**Published:** 2025-12-31

**Authors:** Moon Choi

## Abstract

Transportation services are increasingly digitalized and moving toward automation. As a result, digital skills are essential for accessing and utilizing navigation and rideshare applications, as well as for understanding and making informed choices regarding automated vehicles. However, older adults, often referred to as digital immigrants, face challenges in adopting these technologies, as they emerged later in their lives. This symposium session examines older adults’ adaptation to smart mobility technology, with a particular focus on the digital divide. The session will explore the following key questions: (a) How do older adults perceive safety-related messages of automated vehicle (AV) technologies compared to younger adults? (b) To what extent does training enhance older adults’ ability to use mobile applications for demand-responsive transport (DRT) services? (c) Is a tutorial designed to support older adults with cognitive impairments effective in improving their ability to use navigation and rideshare applications in usability tests? (d) How is the use of navigation technologies associated with perceived constraints and travel behavior among older adults? Discussions on the findings from these four studies will contribute to a deeper understanding of age-related differences in perceptions, digital literacy, and the use of smart mobility technologies. Moreover, the session will provide evidence on the effectiveness of interventions aimed at improving digital literacy related to smart mobility services and their potential impact on the well-being of older adults. This is a collaborative symposium between the Technology and Aging and Transportation and Aging Interest Groups.

